# Clinical Xenotransplantation of Organs: Why Aren't We There Yet?

**DOI:** 10.1371/journal.pmed.0040075

**Published:** 2007-03-27

**Authors:** Muhammad M Mohiuddin

## Abstract

Mohiuddin discusses the lessons learned from large animal xenograft models and why the immunological barrier is still the most important hurdle preventing clinical xenotransplantation of organs.

## Introduction

Organ transplantation across a species barrier—xenotransplantation—has been attempted for over a century. Given the rapidly increasing gap between the organs required and those available for transplantation, over the last 20 years xenotransplantation has been aggressively pursued as a promising supplement to allotransplantation (see Glossary).

Progress in this field from the late eighties to the late nineties had been steady, but shrinking funding, ethical and regulatory issues, threats of transmission of infection, and diminished interest by industry have resulted in a significant decline of enthusiasm in this field. But the recent development of genetically modified pigs that are more compatible with humans has reinstated hope for the success of xenotransplantation of organs. However, whether such genetic modifications are necessary to prevent xenograft rejection of porcine cells is questionable.

## Early Experiments Using Small Animal Models

A significant amount of information about the mechanism of solid organ xenograft rejection was gained from earlier experiments using small animal models. Experimental protocols were successfully generated to induce graft accommodation and donor-specific tolerance, the latter, for example, through the generation of microchimerism [1–6]. In accommodation studies, production of antibodies in transplanted animals was delayed, and when the antibodies were later allowed to return, the transplanted organ had developed a means of protection from these antibodies, thus preventing antibody-mediated rejection [5,7]. In tolerance studies, the immune system of the recipient was manipulated so that it learned to recognize the foreign graft as self [1,3,4,8]. Costimulatory blockade and suppression of T and B cells were also successful in achieving long-term graft survival in small animal models [9–14].

**Glossary**

**Allogeneic:** Two or more strains are stated to be allogeneic to each other when the genes at one or more loci are not identical in sequence in each organism.

**Allotransplantation:** Transplantation of an allograft.

**Autologous:** Derived from the same organism.

**Heterotopic:** Occurring in an abnormal position.

**Microchimerism:** The presence of two genetically distinct and separately derived populations of cells, one population being in low concentration, in the same individual or organ ( e.g., bone marrow).

Thus, work in small animal models of solid organ xenografts clearly showed that xenotransplantation initiates a variety of inflammatory, immune, and coagulation responses, and the successful suppression of these responses encouraged researchers to move to larger animal models. Unfortunately, the task of extending graft survival in large animal models such as pig-to-nonhuman primate (NHP) has proven to be a tall order. The mechanism of rejection is found to be more complex and experiments using large animals have resulted in identification of new pathways responsible for substantial anti-donor xenogeneic responses [15,16].

In this article, I discuss the lessons learned from large animal xenograft models and why the immunological barrier is still the most important hurdle preventing clinical xenotransplantation of organs. I also briefly consider other barriers, such as ethical concerns and concerns about viral disease transmission.

## Alternatives for Overcoming End-Stage Organ Failure

Patients requiring organ transplantation have limited options. For example, total artificial hearts or mechanical devices have great potential for replacing or improving the function of a diseased heart. However, while ventricular devices have helped patients with cardiac failure [17], implantation devices have suffered from thrombotic complications and are not yet proven suitable for replacing transplantation [18].

Autologous adult stem cell transplantation has garnered significant interest over the past few years. This procedure has the potential to repair damage due to myocardial infarction and local defects [19–21]. Allogeneic stem cell transplantation may play a role in delaying the need for transplantation. However, neither of these methods have the potential to replace entire organs.

The idea of growing organs in culture dishes has fascinated scientists for years. Attempts to grow organs (e.g., kidneys) in vitro have yielded small sized organs that lack vascularization [22]. Attempts to grow organs in vivo, in which fetal tissue has been shown to grow into functional organs, have shown some promise. The progress in this field is gradual but I believe that attempts to grow organs are further away from clinical practice than xenotransplantation. Considering all these options, xenotransplantation seems to be one of the most viable and complete options for replacing organs to treat end-stage diseases.

## Mechanisms of Xenograft Rejection in Animal Models

### Antibody-mediated rejection

In experimental xenotransplantation between discordant species, i.e., species that are phylogenetically distant, the graft undergoes hyperacute rejection (HAR) within minutes. In the pig-to-NHP combination, an example of discordant species combination, HAR is primarily mediated by preformed xenogeneic natural antibody (XNA, predominantly IgM) against a galactose residue (Galactose alpha 1,3-Galactose [Gal]) expressed on pig vascular endothelium [23,24]. Gal is expressed by pigs and most other mammals [25,26]. Binding of XNA to Gal leads to activation of the complement cascade, which causes endothelial damage, thrombus formation, and ultimately a very rapid graft rejection within minutes [23,27,28].

If this antibody- and complement-mediated rejection is averted by measures described below, the transplanted organ undergoes delayed xenograft rejection or acute vascular rejection [29]. In these cases, elicited ( IgM and IgG) antibodies recognize Gal and other non-Gal antigens on the vascular endothelium leading to its activation and rejection of xenografts [30].

### Inefficient regulation of homeostasis leading to intravascular coagulation

Intravascular coagulation is triggered by either antibody/cell-mediated damage of the endothelium or by coagulation factor incompatibilities between two species, and plays a significant role in xenograft rejection. On activation by either antibody binding, or directly by T cells, NK cells, or macrophages, endothelium changes from its anticoagulant state to a procoagulant state by up regulation of von Willebrand factor and production of tissue factor leading to thrombus formation, hemorrhage, and rejection of the graft.

The molecular incompatibilities of coagulation and complement systems further contribute to the rejection process in many porcine-to-NHP systems. For example, porcine thrombomodulin cannot bind to NHP thrombin, and therefore cannot activate protein C and prevent thrombosis [31].

### Rejection of Gal knockout pig xenografts

Recently pigs have been generated in which the enzyme 1,3-galactosyltransferase (GT) gene, which encodes the enzyme responsible for adding Gal residues to many cell surface molecules, has been disrupted. When organs from these Gal-deficient pigs are transplanted into baboons, there is no activation of complement due to the binding of preformed anti-Gal antibody and hyperacute rejection is prevented. Nevertheless, antibodies to non-Gal antigens, which can also directly activate endothelium, and persistence of coagulation incompatibilities leads to graft rejection [15]. These non-Gal antigens have not been fully characterized yet and their potential role in xenograft rejection is under investigation. In Gal knockout (KO) pigs, some investigators have shown an alternate mechanism of surface expression of Gal, suggesting that elimination of Gal is not complete [32].

### Cell-mediated rejection

In vitro studies analyzing the human response to porcine antigens indicates that human T cells can directly recognize porcine major histocompatibility complex, swine leukocyte antigen I & II, and can also recognize porcine antigens indirectly in the context of self major histocompatibility complex, human leukocyte antigen [33]. A recent paper by Davila et al. shows the induction of cytotoxic pig-specific CD4+CD28- lymphocytes capable of direct tissue destruction [34]. Multiple NK cell-mediated xenograft rejection pathways also exist and complicate efforts to neutralize the potent anti-xenograft activity of NK cells. Recent studies using *Gal-/-* porcine endothelial cells showed resistance to NK-mediated antibody-dependent cellular cytotoxicity, but susceptibility to direct NK cell lysis [35,36]. Macrophages have also been shown to target and directly destroy islet xenografts [37].

## Large Animal Models of Xenotransplantation: Recent Progress and Limitations

### Cellular (islet) transplantation

The most promising reports have come from transplanting wild type porcine islets in NHP for the treatment of diabetes, where complete reversal of diabetes was shown consistently for over 100 days [38,39]. Much evidence suggests that adult porcine islets, unlike endothelial cells and many other cell types, do not express the Gal epitope, and are not susceptible to XNA-mediated hyperacute rejection.

But there were a few drawbacks in both of these studies [38,39]. First, a much larger number of islets compared to the number used in clinical transplantation of human islets were infused to control hyperglycemic states. Second, the level of immunosuppression used in the NHP studies would be unacceptable in humans. For the effective use of this method in clinics, a more acceptable immunosuppressive regimen will be needed and if a larger dose of xeno islets is required to overcome hyperglycemia, an alternate site, such as the omental pouch, could be considered to avoid compromising liver function. To avoid immune rejection, porcine islets have also been transplanted successfully using alginate encapsulation; while this method offers promise, additional work is needed [40].

### Solid organ transplantation

Mixed results have emerged from experiments using genetically modified pig-to-baboon organ transplantation. To date, only two groups have reported long-term survival (over three months) of heterotopic porcine cardiac xenografts for in baboons. McGregor et al. have shown long-term survival of human CD46 transgenic pig hearts in baboons by using synthetic Gal conjugate (TPC) and strong immunosuppression [41,42]. Kuwaki et al. transplanted Gal KO pig hearts heterotopically into baboon recipients and demonstrated graft survival of over six months with costimulation blockade and immune suppression [43,44]. McGregor et al. have recently reported (unpublished data; World Transplant Congress, Boston, MA) survival of orthotropic cardiac xenografts with the longest graft survival of over 60 days, indicating the ability of pig heart to sustain life of recipient baboon.

Recently developed techniques of vascular thymic transplantation are also a major step towards tolerance induction to xenografted kidneys [45,46]. Successful prolongation of pig kidney graft survival by simultaneous transplantation of pig thymus has also been reported using Gal KO pigs as source animals [47]. However, the extensive immunosuppression used in these experiments significantly increased the mortality rate due to infections.

**Figure 1:**
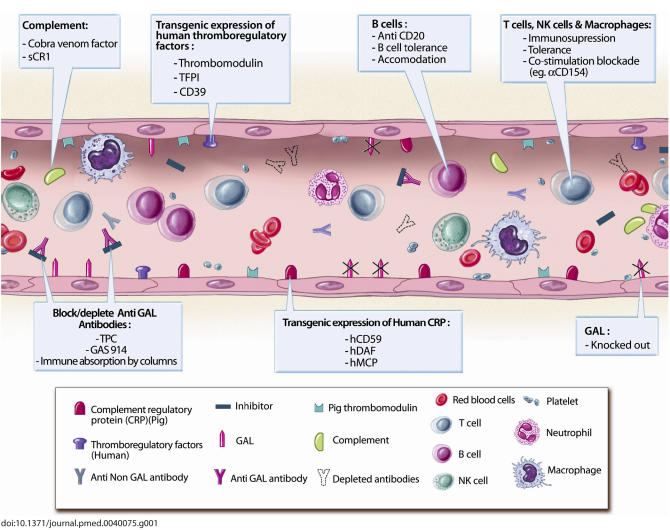
Current Methods to Prevent Xenograft Rejection TFPI, tissue factor pathway inhibitor.

Several other manipulations targeting specific causes of rejection have been developed to overcome xenograft rejection in large animal models. Some of these approaches and their resultant effects are described below (see also Figure 1).

### Targeting antibody-mediated rejection

Investigators have successfully delayed HAR by immunoadsorption of antibody on columns or by blocking the epitope binding with GAS 914 or TPC (Gal-Polyethylene glycol conjugates) [41,48–50]. But reemergence of antibodies had not been properly controlled and still posed a credible problem.

In experiments using Gal-deficient pigs as sources, and in recipients in which anti-Gal antibodies were neutralized, antibodies against non-Gal antigens have induced acute xenograft rejection [15,51]. Kidneys transplanted into baboons from either Gal KO pigs or wild type pigs with Gal neutralized by synthetic Gal conjugates are subject to acute rejection by non-Gal antibodies [15]. The results of this study [15] and studies performed by other groups suggests that when Gal is present, anti-Gal is the primary antibody involved in rejection, but in its absence other non-Gal antibodies play a prominent role in xenograft rejection. Anti-CD20 antibody has been effectively used to eliminate antibody-producing B cells in allotransplantation and in therapies for several leukemias. When this antibody was used for an extended period of time to avoid xenograft rejection, it resulted in serious infections, thus limiting its use to induction therapy. CD20 is not expressed on memory B cells, therefore the antibody against this molecule is unable to suppress anti-pig antibody production by these cells[52].

### Suppressing complement and thromboembolism

Complement is a major contributor to both antibody-mediated rejection and coagulopathies responsible for xenograft rejection. Therefore, measures to prevent complement activation included use of soluble complement receptor 1 (sCR1) [53], cobra venom factor (CVF) [54], and transgenic expression of human complement regulatory proteins such as decay accelerating factor (CD55) [55], CD59 [56], and membrane cofactor protein (CD46) [57]. Expression of decay accelerating factor prevented HAR to some extent [58], but was not sufficient to prevent microangiopathic coagulopathy [59]. Transgenic expression of human CD46 along with the use of strong immunosuppressive agents could partially inhibit graft rejection [42]. Triple expression of human CD55/CD59/alpha 1,2-fucosyltransferase (HT) (alpha 1,2-fucosyltransferase competes with GT for substrate to reduce Gal expression) also averted HAR, but was also not successful in significantly prolonging the graft survival [60].

Drug strategies to prevent thromboembolism included warfarin or low molecular weight heparin [61], aspirin [62], and anti-platelet therapy with aspirin and clopidogrel [63], but none of them were able to significantly prolong graft survival. Genetic modifications to prevent thrombosis include transgenic expression of human tissue factor pathway inhibitor, CD39, or thrombomodulin. Any reports using these transgenic factors in large animal models are yet to be published. Coagulopathy has also been associated with latent porcine cytomegalovirus (pCMV) infections, but early weaning of pigs has prevented activation of pCMV infections, and consumptive coagulopathy was thereby averted [64].

### Costimulation blockade

Anti-CD154 was used by some groups to prolong graft survival [38,65–67], but thromboembolic complications limited the successful use of this agent [68,69]. Other methods of costimulation blockade that work well in inducing tolerance in small xenograft models have not proven successful in prolonging pig-to-baboon cardiac xenograft [67].

Most of the above techniques currently used to over come xenograft rejection are summarized in Figure 1.

## Beyond Immunological Barriers: Concerns about Ethics and Viruses

All the current regimens used to prolong xenograft survival involve vigorous immune suppression leading to an immunocompromised recipient. This immune suppression could result in infection by pathogens not normally associated with human disease or by newly emerging infectious agents. Categories of potential pathogens are discussed elsewhere [70]. The demonstration that porcine endogenous retroviruses (PERVs) infect human cells in vitro has raised concerns about disease transmission by retroviruses through xenotransplantation [71].

But selective breeding techniques may eliminate a large number of potential pathogens, including pCMV, and possibly reduce infectious endogenous retroviruses. Swine that do not express PERVs that are infectious for human cells have been identified [72]. Long-term retrospective studies of patients treated with pig tissues have not found any evidence of PERV infection in any of the patients tested [73,74]. Antibodies against the highly conserved epitopes encoded by the retroviral genome have been shown to neutralize PERV infectivity, suggesting that there may be a basis for producing a PERV vaccine [75].

However, the risk of infection via xenotransplantation could be perceived differently by patients with end-stage heart disease or a patient with kidney failure inadequately controlled by dialysis. New viruses and other pathogens continue to infect human beings. Whether the risk of transmission of these pathogens will increase with xenotransplantation is not yet known. But the risk can be anticipated, and thus prepared for. A long-term careful follow-up of transplanted patients will be required to monitor for infection by latent viruses and other pathogens. A timely intervention would be important to treat the infection and control its spread to other individuals.

Other barriers to clinical xenotransplantation include ethical concerns, including the objection of animal rights proponents to the use and genetic modification of pigs. Commercial considerations (e.g., high cost) and regulatory requirements to ensure safety and the potential for efficacy may also play a significant role in delaying the transition of xenotransplantation from bench to bedside.

## The Next Steps

It is evident from the progress to date that there are several mechanisms of xenograft rejection which still must be overcome and which will require extensive investigations to make xenotransplantation a clinical reality. There are fewer hurdles to worry about in xenogeneic cellular/islet transplantation, which therefore has greater potential for reaching clinics than solid organ xenotransplantation.

For organ xenotransplantation, just replacing one immunosuppressive agent with another may not serve the purpose. Serious attempts should be made to induce tolerance to xenografts, especially for B lymphocyte mediated immunity, or to further modify the genetic makeup of the Gal KO pig to make it less immunogenic in both NHP and humans. More experiments are needed with life supporting organ transplantation to determine the physiologic restrictions of this procedure. Further, cross species transmission of pathogens should be studied in greater depth as this issue will also limit the transition of xenotransplantation to clinics.

With diminishing industry support and limited funding from granting agencies, it has become more evident that finding a solution to xenograft rejection is not within the scope of one investigator or laboratory. Therefore, it is imperative that major groups working on xenotransplantation share their information and expertise to formalize a joint approach to make this unique field a clinical reality.

I believe that the research in this field is progressing in the right direction and, in the course of seeking to solve xenograft rejection, has significantly advanced our understanding of several important immunological mechanisms, including the role of anti-carbohydrate antibodies, memory B cells, coagulation cascades, and cancer therapy. There is a fair amount of optimism that with careful planning and a coordinated effort, the dream of clinical xenotransplantation can be achieved.

## Abbreviations

HAR: hyperacute rejection
KO: knockout
NHP: nonhuman primate
pCMV: porcine cytomegalovirus
PERV: porcine endogenous retrovirus
XNA: xenogeneic natural antibody
